# Inhibitory Effect of Olive Phenolic Compounds Isolated from Olive Oil By-Product on Melanosis of Shrimps

**DOI:** 10.3390/antiox10050728

**Published:** 2021-05-05

**Authors:** Antonio Lama-Muñoz, Antonio Gómez-Carretero, Fátima Rubio-Senent, Alejandra Bermúdez-Oria, Inés Maya, José G. Fernández-Bolaños, Blanca Vioque, Juan Fernández-Bolaños

**Affiliations:** 1 Department of Food Phytochemistry, Instituto de la Grasa (Spanish National Research Council, CSIC), Pablo Olavide University, Building 46, Ctra de Utrera km 1, 41013 Seville, Spain; alama@cica.es (A.L.-M.); fatimars_85@hotmail.com (F.R.-S.); aleberori@ig.csic.es (A.B.-O.); bvioque@ig.csic.es (B.V.); 2 Department of Organic Chemistry, Faculty of Chemistry, University of Seville, Profesor García González 1, 41012 Seville, Spain; angocar@unex.es (A.G.-C.); imaya@us.es (I.M.); bolanos@us.es (J.G.F.-B.)

**Keywords:** Atlantic ditch shrimp, melanosis inhibition, refrigerated storage, olive oil by-product, phenolic antioxidants, synthetic derivatives

## Abstract

Melanosis is an unsolved problem of the crustacean industry and the cause of great loss of value. This study investigates the effect of two potent, natural antioxidants isolated from olive waste (hydroxytyrosol, HT and 3,4-dihydroxyphenylglycol, DHPG) and three novel HT-derivatives containing selenium and sulfur (dihydroxytyrosyl diselenide, *N*-hydroxytyrosyl selenourea, and *N*-hydroxytyrosyl thiourea) on the prevention of melanosis in Atlantic ditch shrimp (*Palaemonetes varians*) during refrigerated storage. These results clearly demonstrate the positive inhibitory effect of DHPG and dihydroxytyrosyl diselenide on delaying melanosis in vivo, although this effect was not dose dependent. The effect was associated with a concomitant-inhibitory effect on tyrosinase activity in vitro. To our knowledge, so far no studies on the prevention of melanosis have been conducted on this small specie of shrimp which is available in large quantities at any time of the year at low cost. Studies with these promising compounds could then be extended to other more economically important species with a greater guarantee of success.

## 1. Introduction

Shrimp and other crustaceans are highly perishable products with limited shelf life due to melanosis. Atlantic ditch shrimp (*Palaemonetes varians*) is a brackish water crustacean with an almost colorless body that is 30–50 mm in length. Despite its small size, this specie is commercially captured with certain local importance. It is frequently harvested for human consumption where it reaches market prices similar to that of the penaeid species and also is used in aquaculture as a live diet for more demanding species [1]. Melanosis is a natural post-mortem process caused by the polymerization of phenols into insoluble black pigments, melanins. This phenomenon is triggered by a biochemical mechanism involving the oxidation of phenols by tyrosinase, an enzymatic complex found in almost all organisms. Tyrosinase (also called polyphenol oxidase, PPO), catalyzes two successive reactions involving molecular oxygen: (1) the hydroxylation of monophenols leading to the formation of *o*-diphenols, and (2) the subsequent oxidation into *o*-quinones that in turn polymerize into brown, red, or black pigments [2]. Although the melanosis of shrimp is unsightly, it does not pose a risk to the consumer’s health. However, melanosis drastically reduces the value of the products on the market, causing significant economic losses [3]. This deteriorative process can be delayed by freezing, refrigeration, or storage of the shrimp on ice. However, this reaction cannot be totally prevented, and melanosis must be controlled or eliminated by other means [4,5].

Melanosis in crustaceans is normally controlled by reducing agents such as sulfites and their derivatives. However, as sulfites can provoke severe allergic reactions, alternative compounds have also been intensively studied. Thus, 4-hexylresorcinol [5,6] and kojic acid [7] have been widely applied. Other safer compounds, such as ficin enzyme [8], citric acid [5], and dodecyl gallate [9], could potentially find applications in this sense. More recently, 3-hydroxypyridinone-l-phenylalanine conjugates [10], cinnamon essential oil [11], and thiol compounds, such as cysteine and glutathione [12], have been extensively reported to be effective. Recent studies have also shown that combined effects of freezing and modified atmosphere packaging could be applied without chemicals to reduce blackspot in shrimp [13]. 

Plant phenolic compounds are also promising agents to be used as shrimp preservatives. Ferulic acid and catechin were reported to be very effective in controlling melanosis in Pacific white shrimp [14,15]. Extracts of grape seeds, tea, and pomegranate peel have high polyphenol content and inhibit the melanosis of shrimp [16,17].

In olive fruit, two important phenolic compounds in terms of their beneficial biological properties are hydroxytyrosol (HT) and 3,4-dihydroxyphenylglycol (DHPG). Both phenols are found free and as secoiridoid derivatives in virgin olive oil [18], olive drupes [19], as well as in olive waste (called alperujo), a by-product of the olive oil extraction [20]. HT is considered a functional food and has been granted the GRAS (generally recognized as safe) status by the FDA (U.S. Food and Drug Administration) [21]. Furthermore, the European Food Safety Authority (EFSA) has endorsed the health claim that the consumption of HT and its derivatives (> 5 mg/day) protects LDL particles from oxidative damage [22].

On the other hand, some carbohydrates containing selenium [23], selenourea [24], and 1,3-selenazol derivatives [25], as well as organic sulfur derivatives such as glutathione (GSH), l-cysteine, *N*-acetyl-l-cysteine, and thioureas [12], have also shown to inhibit the tyrosinase activity. 

**Figure 1:**
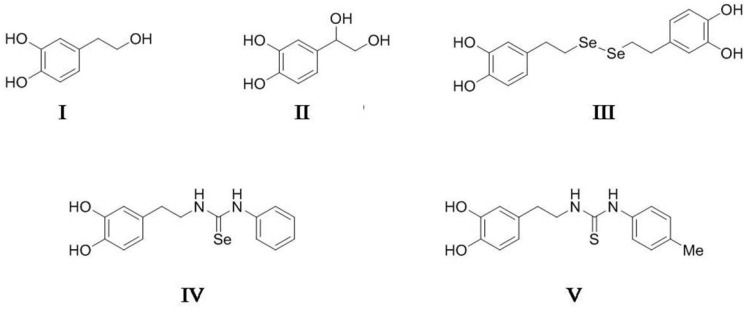
Chemical structures of hydroxytyrosol (HT) (**I**), 3,4-dihydroxyphenylglycol (DHPG) (**II**), dihydroxytyrosyl diselenide (diselenide of bis-HT) (**III**), *N*-hydroxytyrosyl selenourea (selenourea) (**IV**), and *N*-hydroxytyrosyl thiourea (thiourea) (**V**).

Currently, no information exists regarding the use of HT (I) and DHPG (II) isolated from olive by-products nor of certain novel HT-derivatives containing selenium or sulfur, such as dihydroxytyrosyl diselenide (diselenide of bis-HT) (III), *N*-hydroxytyrosyl selenourea (selenourea) (IV), and *N*-hydroxytyrosyl thiourea (thiourea) (V) (Figure 1 and Supplementary Materials), on the prevention of melanosis in crustaceans. The aim of this study was to analyze the effect of these compounds on the prevention of melanosis of Atlantic ditch shrimp (*Palaemonetes varians*) during refrigerated storage and to determine the potential inhibitory effect on tyrosinase activity.

## 2. Materials and Methods

### 2.1. Shrimp Collection

Fresh Atlantic ditch shrimp (*Palaemonetes varians*) collected in the Guadalquivir estuary (Sanlúcar de Barrameda, Cádiz, Spain) were purchased from a local market at different seasons of the year. The shrimp were transported alive on ice to the laboratory, and the experiments were carried out in the next 2–4 h.

### 2.2. Isolation of HT and DHPG from Olive Oil By-Product

HT and DHPG were extracted by hydrothermal treatment at 50–80 °C for 60 min from alperujo, the by-product of the olive oil extraction in the two-phase separation system. This by-product was collected from a virgin oil mill (Almazara Experimental, Instituto de la Grasa, Seville). After the treatment, a liquid phase rich in both compounds was obtained. HT and DHPG were purified by chromatography fractionation with a yield of 90–95%. These processes have been described and patented by Fernández-Bolaños et al. [26].

### 2.3. Preparation of Novel Phenolic Derivatives Containing Selenium and Sulphur

The synthesis of diselenide of bis-HT (III) was carried out from HT (I) via hydroxytyrosyl bromide (VI) (Figure S1 in Supplementary Materials). The bromide-derivative (VI) was prepared in 80% yield using a modified version of Pluempanupat’s et al. conditions [27], by treatment of (I) with PPh_3_ and CBr_4_ (1:3:2) using DMF containing one equivalent of sodium ascorbate to avoid the oxidation of the catechol moiety. Acetylation of (VI) followed by nucleophilic displacement of the bromide of (VII) with in situ generated NaHSe in ethanol containing solid CO_2_ to prevent deacetylation furnished dimeric diselenide (VIII) in a 47% yield for the two steps. Deprotection of (VIII) with K_2_CO_3_ in methanol/dichloromethane (1:1) (*v/v*) afforded (III) in a 51% yield [28]. 

Selenourea (IV) and thiourea (V) were prepared in one step by the coupling of dopamine (IX) with non-commercial phenyl isoselenocyanate [29] and commercial p-tolyl isothiocyanate, respectively (Figure S2 in Supplementary Materials). The identification of all synthesized compounds is shown in Supplementary Materials.

### 2.4. Treatment of the Shrimp

Several dipping solutions were prepared by dissolving the active compounds in distilled water. The concentration of the different solutions (between 1 µM and 5 mM) varied for each experiment according to the bibliography and the assays carried out for inhibiting the tyrosinase activity (data not shown). The number and size of the shrimps also varied because they were collected at different seasons of the year. While in the first experiment, bigger shrimp could be selected, in the rest experiments, it was not possible because the shrimp were smaller and homogeneous. The immersion times were 30 min for the olive-active compounds, although in the third experiment, lower times were also assayed with DHPG (5, 10, and 15 min) (data not shown). As a result of this assay, immersion times of 10 min were chosen for the synthetic active compounds. After dipping, the shrimps were drained for 5 min at room temperature (19–22 °C), placed on a ceramic container, overwrapped with polyvinyl chloride films, and stored at 4–6 °C. Shrimps dipped in distilled water were used as control treatments. During storage, the formation of black spots on the shrimp shell was determined at different times by color measurements.

### 2.5. Treatments with HT and DHPG

In the first experiment carried out in November, lots of 15 g of shrimps corresponding to 37–68 shrimps per lot were selected for their bigger size (0.32 ± 0.06 g average weight ± SD). Each lot was immersed for 30 min in 100 mL of dipping solution containing HT at 0.1, 0.5, 1.0, and 5.0 mM or DHPG at 0.1, 0.5, 1.0, 2.5, and 5.0 mM. Black spot formation was observed after 6 days at 4–6 °C. In the second experiment carried out in April, lots of 50 g of shrimps corresponding to 323–415 shrimps per lot (0.14 ± 0.01 g average weight ± SD) were immersed for 30 min in 100 mL of dipping solution containing HT at 10, 50, and 100 µM, DHPG at 10, 50, 100, and 500 µM, or in a mixture of HT and DHPG (1:1) (*v/v*) (50 µM each). The formation of black spots on the shrimp shell was observed after 3 days at 4–6 °C. In the third experiment carried out in September, lots of 60 g of shrimps corresponding to 215–403 shrimps per lot (0.20 ± 0.03 g average weight ± SD) were tested. Each lot was immersed for 30 min in 100 mL of dipping solution containing HT or DHPG at 10, 25, 50, and 75 µM, or in a mixture of ascorbic and citric acids (1:1) (*v/v*) (50 µM each) as A reference solution. In this experiment, the shrimps were also immersed in 100 mL DHPG 50 µM for lower storage times (5, 10, and 15 min) (data not shown). The color measurements were performed after 5 days at 4–6 °C.

### 2.6. Treatments with Diselenide of bis-HT

The effect of diselenide of bis-HT, a novel HT-derivative containing selenium (Figure 1, Figures S1 and S2), on the melanosis formation of shrimp was also analyzed. This compound was assayed at 0.5, 1, 10, and 20 µM, and the effect was compared with HT and DHPG at 10, 50, and 75 µM. This experiment was carried out in November with an immersion time of 10 min. Lots of 35 g of shrimps corresponding to 101–133 shrimps per lot (average weight of 0.29 ± 0.02 g) were immersed in 100 mL of each dipping solution. The shrimps were analyzed after 2 and 5 days of refrigerated storage (4–6 °C).

### 2.7. Treatments with Selenourea and Thiourea

The effects of two novel phenolic derivatives containing selenium and sulfur in their structures (Figure 1, Figures S1 and S2) on the melanosis formation of shrimp were also investigated. Selenourea and thiourea were assayed at 1, 10, and 20 µM and their effects compared with DHPG at 20, 50, and 75 µM. In this experiment carried out in February, lots of 40 g of shrimps corresponding to 156–177 shrimps per lot (average weight of 0.24 ± 0.01 g) were immersed for 10 min in 100 mL of each dipping solutions. The formation of black spots on the shrimp shell was observed after 2 and 5 days of refrigerated storage (4–6 °C).

### 2.8. Determination of Melanosis Formation in Atlantic Ditch Shrimp

The melanosis or blackening of the shrimp samples was evaluated by inspection of the color measurements by five trained panelists. Since the shrimps were very small, a three-criteria scoring test was used for assessing the degree of pigmentation and number of shrimps affected: (1) low pigmentation, complete absence of black spots, or a few small black spots (less than 10% of the shrimp surface affected); (2) medium pigmentation, presence of spotting on the carapace (between 10 and 40% of the shrimp surface affected, head and body); and (3) high pigmentation, considerable or substantial spotting over the entire shrimp (up to 40% of the shrimp surface affected, head, body, and tail).

### 2.9. Inhibition of Tyrosinase Activity

The inhibition was tested according to the method of Prasad et al. [30] with some modifications. The inhibition of tyrosinase activity is accompanied by a decrease in the formation of products that absorb at 490 nm. This decrease is indicative of a decrease in the formed product and hence a greater inhibition of the enzyme activity. The mushroom tyrosinase used for this bioassay was purchased from Sigma-Aldrich (St. Louis, MO, USA), and l-tyrosine was used as a substrate for the monophenolase assay. Fifty μL of 5 mM l-tyrosine was added to 100 μL of 50 mM phosphate buffer pH 6.6 in a 96-well microplate. After 10 min at 30 °C, 50 μL of the sample solution and 50 μL of an aqueous solution of tyrosinase (50 Units/mL) were added to the mixture. The absorbance at 490 nm was recorded every 20 s for 15 min using a microplate reader (Bio Rad iMark^TM^, Hercules, CA, USA). or tyrosinase-inhibitory activity, a time point of 300 s was fixed and a blank without l-tyrosine was included for each sample. The absorbance of substrate and tyrosinase was set at 100% oxidation. The tyrosinase-inhibitory activity was expressed as a percentage and was calculated using the following formula:

Absorbance (*A*) of blank − *A* of sample/*A* of blank × 100 (1)

### 2.10. Statistical Analysis

IBM SPSS software (Armonk, NY, USA) was used for statistical analysis. Comparisons amongst samples were made using the F-test in a one-way analysis of variance (ANOVA table). A *p*-value ≤ 0.05 was considered significant. Average percentages of the surface affected were used for statistical analysis as 5, 25, and 75% of the shrimp surface affected for low, medium, and high, respectively. 

## 3. Results and Discussion

### 3.1. Effects of HT and DHPG on Melanosis Formation of Atlantic Ditch Shrimp

An initial experiment with shrimps caught in November selected for their bigger size was performed (Table 1, Experiment 1). After six days of refrigerated storage at 4–6 °C, 65% of the shrimp in the control group developed an extensive black area and were classified as high pigmentation, 10% had medium pigmentation, while the remaining 25% lacked black spots on the head or had very few or were pale in color and thus were regarded as low pigmentation. In the samples treated with HT and DHPG, a clear decrease in the percentage identified as high pigmentation was observed with insignificant melanosis for three of the DHPG concentrations tested. Samples treated with DHPG at 0.1, 0.5, and 1.0 mM showed a very significant increase in the percentage considered as low pigmentation: up to 70–76% compared to 25% of the control group. However, these percentages decreased considerably when higher concentrations of the active compounds were applied (2.5 and 5.0 mM of DHPG). The statistical analysis revealed significant differences in respect to the control for all concentrations of the active compounds applied except for the highest one of HT of 5 mM. The most significant results were obtained for DHPG at the lowest concentrations tested: 0.1 and 0.5 mM. Therefore, the delay of melanosis in shrimp treated with HT and DHPG does not follow a dose-dependent pattern. Similar results were reported with Pacific white shrimp treated with green tea extract. In these studies, the control on the formation of melanosis was more effective in shrimp treated at 5 g/L than in those treated at 10 g/L [17]. These concentrations are considerably higher than the effective concentrations now reported for the olive phenolic compounds. Furthermore, in our assay conditions, DHPG showed greater efficacy in controlling melanosis than HT at similar concentration levels. Following HT, there was a notable increase in the percentage of shrimp with a medium appearance. These facts also coincided with the development of a certain reddish coloration at high concentrations of phenol (Table 1, Experiment 1).

In the second experiment, the treatments were carried out on shrimps caught in April with an average of 368 shrimps per treatment. In this case, a narrower range of concentrations closer to the effective concentrations determined for the first experiment was applied. HT was assayed at the range from 10 µM to 100 µM and DHPG at the range from 10 µM to 500 µM. A mixture of HT and DHPG (1:1) (*v/v*) (50 µM each) was also assayed. The results given by the panelists after three days of refrigerated storage at 4–6 °C are shown in Table 1, Experiment 2. As in the first experiment, DHPG was shown to be more effective in controlling melanosis than HT. For both antioxidants, the inhibitory effect was not dose-dependent and maximal reduction in the formation of melanosis was obtained at 50 µM by both active compounds. However, at this concentration, DHPG tripled the percentage of shrimps considered as low pigmentation in respect to the control, while HT only increased it 1.6-fold. On the other hand, a mixture of both antioxidants did not substantially improve the results obtained separately. For instance, at 100 µM of HT, a similar reduction of the percentage of shrimps considered high pigmentation was obtained in respect to the combination HT/DHPG at 50 µM each. This reduction was concomitant with the increase in the percentage considered medium pigmentation and also coincided with the reddish tone coloration yet described in the first experiment. Statistical differences were obtained by 100 µM HT and by all the tested concentrations of DHPG. The best results were again at lower concentrations (Statistical differences were obtained at 100 µM HT, and at all the tested concentrations of DHPG. The best results were obtained again at the lower concentrations). (Table 1, Experiment 2).

For the third experiment, the shrimps caught in September were immersed for 30 min in different low concentrations of both phenols. A mixture of ascorbic and citric acids was also assayed as a positive reference. L-ascorbic acid can reduce *o*-quinones in the original diphenols, and citric acid helps to chelate copper present in the active site of the responsible enzymes [6]. The results after five days of refrigerated storage at 4–6 °C are shown in Table 1, Experiment 3. In this experiment, all treated samples exhibited less black spot formation than in the untreated one, in which only 5% of the shrimps were considered low pigmentation. In terms of the different treatments applied, only slight differences between samples, including those treated with the acids mixture, were observed. All treatments showed better results than the control except for the DHPG at 25 µM. The best results corresponded again to 50 µM of DHPG which was significantly better than those of the control and of the positive reference of the acids mixture. For this DHPG concentration, immersion times between 5 and 10 min were sufficient to lower the formation of melanosis compared to 15 and 30 min (data not shown).

### 3.2. Effect of Diselenide of bis-HT on Melanosis Formation of Atlantic Ditch Shrimp

**Figure 2:**
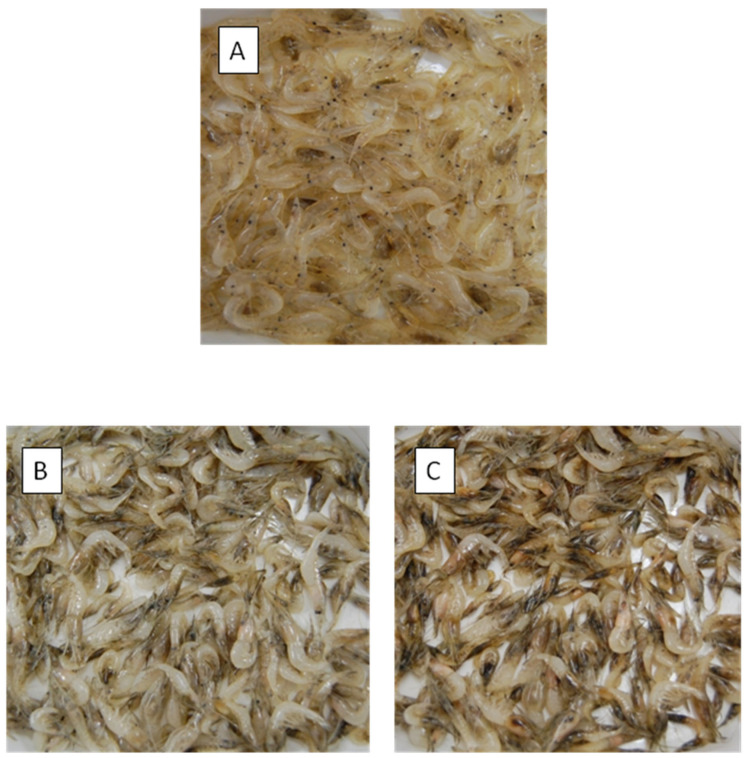
Atlantic ditch shrimp treated for 10 min with 10 µM of diselenide of bis-HT. (**A**) shrimp before the treatment, (**B**) treated shrimp after 5 days of refrigerated storage at 4–6 °C, (**C**) untreated shrimp after 5 days of refrigerated storage at 4–6 °C.

The effect of diselenide of bis-HT (III) (Figure 1 and Supplementary Materials), a novel HT-derivative containing selenium, was also studied in order to avoid melanosis. This synthetic HT-derivative with a reducing power five-fold higher than HT and an antiradical activity two-fold higher (unpublished results), was assayed at the range from 0.5 µM to 20 µM. The effect of this compound was compared to those of HT and DHPG at the range of 10 µM to 75 µM. In this experiment, lots of shrimp with an average of 122 shrimps were immersed for 10 min in the different treatments. The melanosis scores after two and five days of refrigerated storage at 4–6 °C are illustrated in Table 2 and Figure 2.

In control samples, the melanosis increased continuously with the storage time. The extent of melanosis was noticeably decreased in samples treated with diselenide of bis-HT at the range of 1 µM to 20 µM after two days of refrigerated storage. The immersion of the shrimps at the lowest concentration level of the active compound (0.5 µM) did not reduce the black spot formation of melanosis, with significant differences found in respect to the control at the rest of the concentration levels. The best results for this novel synthetic compound were obtained for 1 and 20 µM. For 1 µM of diselenide of bis-HT, the effect was quite similar to that obtained for 10 µM of HT and for 10 and 50 µM of DHPG. However, after five days of refrigerated storage, the melanosis increased substantially with all the treatments. Significantly lower levels of melanosis were obtained in samples treated with 50 µM DHPG followed by those treated with 10µM diselenide and with 50 µM HT (Table 2).

### 3.3. Effects of Selenourea and Thiourea on Melanosis Formation of Atlantic Ditch Shrimp

The effects of selenourea (IV) and thiourea (V) (Figure 1 and Supplementary Materials) on the formation of melanosis of shrimp in comparison with the effect of DHPG were also investigated. Selenourea and thiourea have antioxidant activities between two- and four-fold higher than HT and DHPG, respectively (unpublished results). In this experiment, an average of 167 shrimps per treatment was used and the results are shown in Table 3.

The onset of melanosis in the control group was quite similar to that of previous assays. After two days of refrigerated storage, the shrimps treated 10 min with 10 µM of selenourea showed the best appearance, with a double percentage of shrimps considered as low pigmentation in respect to the control. However, after five days of refrigerated storage, the melanosis scores in the control sample as well as in the samples treated with selenourea and thiourea were quite similar, with significant differences found only for the highest concentration of both compounds (20 µM). The statistical analysis revealed that after two days of refrigerated storage, the best records were achieved by 10 and 20 µM of selenourea followed again by 50 µM of DHPG. However, after five days of storage, 50 µM of DHPG was again the best. These quite reproducible results indicated that 50 µM of DHPG was the most effective condition, among those tested, for delaying melanosis of this small specie of shrimp.

### 3.4. Inhibition of Tyrosinase Activity

**Figure 3:**
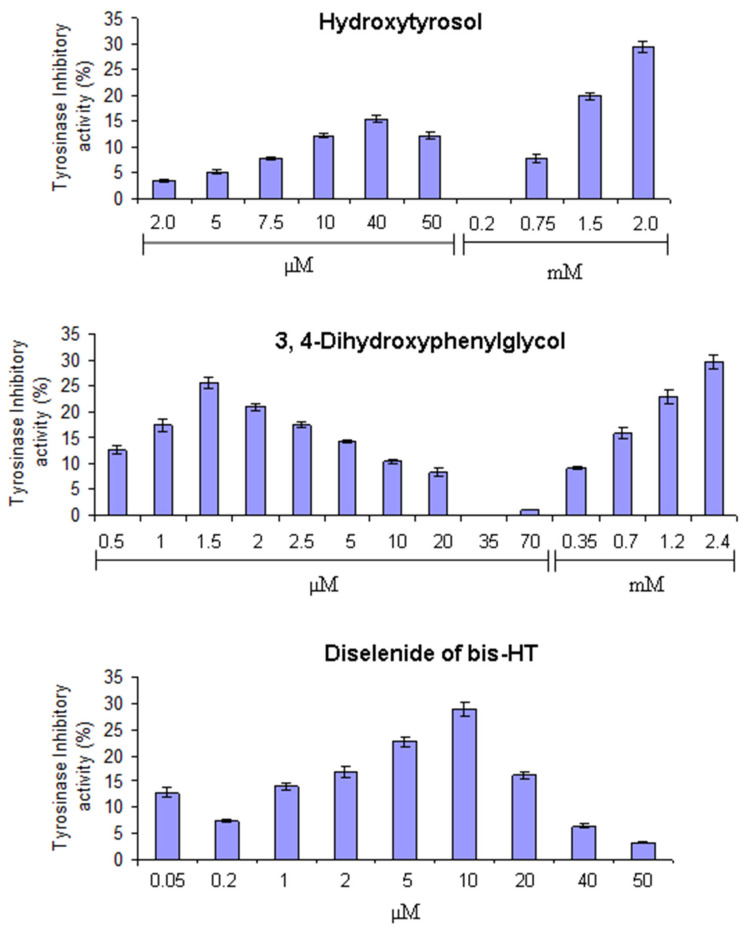
Tyrosinase-inhibitory activity (%) of hydroxytyrosol (HT), 3,4-dihydroxyphenylglycol (DHPG), and dihydroxytyrosyl diselenide (diselenide of bis-HT). Bars represent standard deviations (n = 3). The values showed significant differences (*p* ≤ 0.05) for all tested concentrations except for 0.05 and 1 µM of diselenide of bis-HT.

The inhibitory effects of HT, DHPG, and diselenide of bis-HT on tyrosinase activity are shown in Figure 3. Maximum effects (over 30%) were obtained for HT and DHPG at the highest concentration levels tested (ca 2 mM), with significant increases with 0.75 mM for HT and with 0.35 mM for DHPG. Moreover, both phenols inhibited tyrosinase activity in a non-concentration-dependent manner exhibiting a second substantial maximum at lower concentrations. DHPG showed the highest percentage of inhibition (26%) at 1.5 µM as compared to 15% for HT at 40 µM. These results clearly indicated that the inhibitory effect of DHPG on tyrosinase activity is considerably higher than that of HT. Diselenide of bis-HT showed the highest antityrosinase activity (29%) at 10 µM which decreased up to 3% at 50 µM. Like HT and DHPG, diselenide of bis-HT did not show an inhibitory effect in a dose-dependent manner. At concentrations between 1 and 2 µM, DHPG did possess higher anti-tyrosinase activity than HT and diselenide of bis-HT (Figure 3).

Thus, the most effective compound in retarding the melanosis of shrimp was DHPG. DHPG also exhibits the greatest anti-tyrosinase activity at the lower concentration levels. These results could indicate that the additional hydroxyl group in the benzylic position could be responsible for the inhibitory effect because of the hydrogen bonding. In fact, hydroxyl groups of certain phenolic compounds form hydrogen bonding with the active site of tyrosinase, resulting in steric hindrance or altered conformation that could lower the enzyme activity [31].

However, the reasons for which HT, DHPG, and diselenide of bis-HT retard the appearance of black spots on the shrimps, causing tyrosinase inhibition, remain unclear. Their antioxidant activities could well explain both effects. HT, DHPG, and diselenide of bis-HT are potent antioxidants with strong reducing power and free-radical scavenging activity [32,33]. Thus, they might reduce *o*-quinones derived from the formation of dopa and, as a consequence, melanosis could be delayed. The structural similarity of the *o*-diphenol compounds HT, DHPG, and diselenide of bis-HT to tyrosinase substrates such as tyrosine and L-dopa should also be considered. Oxidation of 1 mM HT was demonstrated in the presence of mushroom tyrosinase, which catalyzes the aerobic conversion of *o*-diphenols to quinones [34]. However, from our results, it is not clear if the tested compounds might be acting as inhibitors or as substrates for the monophenolase activity.

### 3.5. Use of Atlantic Ditch Shrimp as Crustaceans Model System for Preliminary Studies on Melanosis

These studies demonstrate for the first time that this small-bodied crustacean, or other closely related species, could be used as low-cost model systems for preliminary studies on melanosis that investigate the effects of potential inhibitors. The results obtained using these species of small crustaceans could be extended to other species of greater economic importance and during longer storage times. In addition, these preliminary studies with easily obtainable species could be also used to evaluate the commercial viability of the different treatments. 

Melanosis is a significant problem for the crustacean industry and the cause of loss of value. Experiments with large crustaceans, such as lobster, crab, tiger prawn, Pacific white shrimp, or deep-water pink shrimp, allow for better colorimetric evaluation but generally are restricted to few individuals per assay due to their high price and limited availability [4,5,17]. Color, head and body dislocation, odor, texture, and appearance of black spots are more easily assessed when the crustaceans are bigger in size. However, in our model system, and despite the reduced scale used which limits statistical value, the results of these five experiments in which each panelist randomly evaluated multiple lots of shrimps have been fairly repetitive. 

Differences may occur among shrimp species and also among their developmental stages at the beginning of storage, which might be coincided with the molting cycle of the shrimps when the progression of melanosis is faster [4]. In this sense, studies carried out on deep-water rose shrimp (*Parapenaeus longirostris*) provide evidence that seasonal changes and physiological factors related to sex, size of the specimen, and spawning period may alter PPO activity and black spot in crustacean decapods [35]. For these reasons, we obtained data using a large number of shrimps for each treatment, which were caught at different seasons of the year. Finally, this model system could be completed with a simple assay of tyrosinase activity-inhibition which can be well correlated with the delay of melanosis. To this end, further research on the isolation of tyrosinase from crustaceans would be necessary.

## 4. Conclusions

The present work reveals the potential use of HT and especially DHPG and diselenide of bis-HT to prevent melanosis in Atlantic ditch shrimp (*Palaemonetes varians*) during refrigerated storage. These preliminary investigations constitute a solid base for later studies in other species of crustaceans of greater economic importance with a higher probability of success. We report that natural antioxidants such as HT and DHPG isolated from olive waste could be used as more effective and safer alternatives than sulfites to maintain quality in shrimp and crustaceans.

## Figures and Tables

**Table 1:** Melanosis score evaluated as degree of pigmentation (low, medium, and high) during refrigerated storage at 4–6 °C of shrimps treated for 30 min with different concentrations of hydroxytyrosol (HT), 3,4-Dihydroxyphenylglycol (DHPG), and a mixture (1:1) of ascorbic and citric acids (AA/CA) ^a^.

			Degree of Pigmentation (%)			
	Treatment		Low	Medium	High	Total of Shrimps
Exp. 1 6 days	Control		25	10	65 ^a^	40
	HT	0.1 mM	19	51	30 ^b^	37
		0.5	0	87	13 ^bc^	53
		1.0	0	82	18 ^bc^	68
		5.0	0	70	30 ^a^	50
	DHPG	0.1 mM	76	24	0 ^e^	50
		0.5	75	25	0 ^de^	43
		1.0	70	26	4 ^d^	51
		2.5	36	22	42 ^b^	40
		5.0	17	48	35 ^b^	46
Exp. 2 3 days	Control		15	51	34 ^a^	387
	HT	10 μM	18	57	25 ^ab^	415
		50	24	48	28 ^ab^	357
		100	11	71	18 ^bc^	387
	DHPG	10 μM	33	48	19 ^c^	346
		50	49	42	9 ^d^	341
		100	17	57	26 ^ab^	380
		500	17	65	17 ^bc^	377
	HT/DHPG	50 µM each	15	68	17 ^bc^	323
Exp. 3 5 days	Control		5	27	68 ^a^	332
	HT	10 µM	16	42	41 ^c^	403
		25	14	34	52 ^bc^	352
		50	18	38	44 ^c^	215
		75	14	40	46 ^c^	301
	DHPG	10 µM	23	35	42 ^c^	317
		25	16	19	65 ^ab^	310
		50	17	56	27 ^d^	253
		75	16	39	44 ^bc^	295
	AA/AC	50 μM each	17	23	60 ^bc^	287

^a^ Different letters (a–c) (a–e) indicate significant difference among groups at *p* ≤ 0.05 for each of the experiments.

**Table 2:** Melanosis score evaluated as degree of pigmentation (low, medium, and high) during refrigerated storage at 4–6 °C of shrimps treated for 10 min with different concentrations of diselenide of bis-HT, hydroxytyrosol (HT), and 3,4-Dihydroxyphenylglycol (DHPG) ^a^.

Treatment		Degree of Pigmentation (%)						
		2 Days			5 Days			Total of Shrimps
		Low	Medium	High	Low	Medium	High	
Control		36	43	21 ^A^	9	17	74 ^a^	130
Diselenide of bis-HT	0.5 µM	31	44	26 ^AB^	21	30	49 ^bc^	119
	1	61	37	2 ^G^	17	13	69 ^ab^	120
	10	52	40	8 ^CD^	27	27	46 ^cd^	125
	20	67	29	4 ^G^	19	27	54 ^bc^	124
HT	10 µM	57	34	8 ^DEF^	18	26	56 ^bc^	132
	50	46	48	6 ^CD^	19	47	34 ^de^	120
	75	32	43	26 ^AB^	10	28	62 ^ab^	101
DHPG	10 µM	60	35	4 ^F^	20	16	64 ^ab^	123
	50	62	31	6 ^EF^	33	33	34 ^ef^	116
	75	47	39	14 ^C^	24	37	39 ^d^	128

Different letters (a–f) indicate significant difference among groups at *p* ≤ 0.05 for each of experiments. Capital letters (A–F) (A–G) for 2 days and lower letters (a–f) for 5 days.

**Table 3:** Melanosis score evaluated as degree of pigmentation (low, medium, and high) during refrigerated storage at 4–6 °C of shrimps treated for 10 min with different concentrations of selenourea, thiourea, and 3,4-Dihydroxyphenylglycol (DHPG) ^a^.

Treatment		Degree of Pigmentation (%)						
		2 Days			5 Days			Total of Shrimps
		Low	Medium	High	Low	Medium	High	
Control		41	55	5 ^A^	14	34	52 ^a^	165
Selenourea	1 µM	48	46	6 ^B^	14	43	44 ^ab^	177
	10	83	15	2 ^F^	13	43	44 ^ab^	156
	20	71	27	2 ^EF^	16	45	39 ^bc^	176
Thiourea	1 µM	49	41	10 ^A^	18	32	50 ^a^	172
	10	43	51	6 ^A^	8	50	42 ^ab^	159
	20	56	40	4 ^CD^	13	52	34 ^bc^	173
DHPG	20 µM	57	39	4 ^CD^	13	50	37 ^bc^	162
	50	65	29	5 ^D^	32	34	34 ^c^	162
	75	45	51	4 ^BC^	22	32	46 ^ab^	164

Different letters (a–c) indicate significant difference among groups at *p* ≤ 0.05 for each of experiments. Capital letters (A–F) for 2 days and lower letters (a–c) for 5 days.

## Data Availability

Data is contained within the article or supplementary materialThe data presented in this study are available in [insert article or supplementary material here.

## Supplementary Materials

The following are available online at https://www.mdpi.com/article/10.3390/antiox10050728/s1, Figure S1: Scheme of synthesis of diselenide of bis-HT, Figure S2: Scheme of synthesis of selenourea and thiourea.
