# Supplementary material for: Inhibitory Effect of Olive Phenolic Compounds Isolated from Olive Oil By-Product on Melanosis of Shrimps
Source: Antioxidants (Basel). 2021 May 5;10(5):728. doi: 10.3390/antiox10050728 (PMC8147989; doi:10.3390/antiox10050728)
Supplement: Supplementary file 1 [file antioxidants-10-00728-s001.zip › antioxidants-1176233-supplementary.pdf]

antioxidants

MDPI

Article

Inhibitory Effect of Olive Phenolic Compounds Isolated from Olive Oil By-Product on Melanosis of Shrimps

Supplementary Materials

Preparation of HT-derivatives

General chemical procedures

Melting points were recorded on an Electrothermal apparatus and are uncorrected. IR spectra were recorded on a Jasco FTIR-4100 instrument. ^{1}H and ^{13}C NMR spectra were recorded on Bruker Avance-300 and Avance-500 spectrometers. The assignments of ^{1}H and ^{13}C signals were confirmed by homonuclear COSY and heteronuclear 2D correlated spectra. Mass spectra were recorded on a Micromass AutoSpec-Q mass spectrometer. TLC was performed on aluminium pre-coated sheets (E. Merck Silica Gel 60 F_{254}), spots were visualized by UV light and by charring with 10% H_2SO_4 in EtOH. Column chromatography was performed using Merck Silica Gel 60 (40–63 µm).

4-(2-Bromoethyl)benzene-1,2-diol (**6**)

To a solution of hydroxytyrosol **1** (500 mg, 3.25 mmol) in DMF (10 mL) at 0 ºC, PPh_3 (2.688 g, 9.74 mmol, 3 equiv), CBr_4 (1.958 g, 6.49 mmol, 2 equiv) and sodium ascorbate (643 mg, 3.25 mmol, 1 equiv) were added. The reaction mixture was stirred under Ar for 1 h at 0 ºC, and for 7 h at rt. Then, the solvent was removed under vacuum and the residue was purified by column chromatography (AcOEt–hexane 1:20 → AcOEt–hexane 1:5) to give **6** as a white solid. Yield 564 mg, 80%; R_F 0.28 (1:2 EtOAc–hexane); mp 102–104 ºC; FT-IR ν_{max} (KBr) ν_{max} 3439, 3323, 2926, 1617, 1531, 1449, 1375, 1269, 1210, 1122,

944, 863, 820, 790, 760 cm^{-1}; ^{1}H-NMR (300 MHz, $CDCl_3$) δ 6.81 (d, 1H, $J_{5,6}$ 8.0 Hz, H-6), 6.73 (d, 1H, $J_{3,5}$ 1.8 Hz, H-3), 6.65 (dd, 1H, $J_{5,6}$ 8.0 Hz, $J_{3,5}$ 1.8 Hz, H-5), 5.20 (brs, 2H, OH), 3.51 (t, $J_{1',2'}$ 7.6 Hz, 2H, CH_2Br), 3.04 (t, $J_{1',2'}$ 7.6 Hz, 2H, CH_2Ar) ppm; ^{13}C-NMR (75.5 MHz, $CDCl_3$) δ 143.6 (C-2), 142.3 (C-1), 132.1 (C-4), 121.2 (C-5), 115.8 (C-6), 115.5 (C-3), 38.7 (CH_2Ar), 33.2 (CH_2Br) ppm; EI-MS *m/z* 346 ([M]$^+$, 27%); HREI-MS *m/z* calcd for $C_8H_9{}^{79}BrO_2$ ([M]$^+$): 215.9786, found 215.9793.

1,2-Diacetoxy-4-(2-bromoethyl)benzene (7)

A solution of **6** (250 mg, 0.856 mmol) in a mixture of Ac_2O–Py (4 mL, 1:1, v/v) was kept at 4 ºC for 14 h. Then, the solvent was removed under vacuum, co-evaporating with toluene and ethanol, and the residue purified by column chromatography (AcOEt–hexane 1:20→AcOEt–hexane 1:5) to give **5** as a yellowish oil. Yield 237 mg, 92%; R_F 0.42 (AcOEt–hexane 1:2); FT-IR ν_{max} (KBr) 2925, 2848, 1766, 1591, 1507, 1427, 1368, 1256, 1203, 1175, 1107, 1010, 901 cm^{-1}; ^{1}H-NMR (300 MHz, $CDCl_3$) δ 7.14 (d, 1H, $J_{5,6}$ 8.1 Hz, H-5), 7.10 (d, 1H, $J_{3,5}$ 1.7 Hz, H-3), 7.06 (dd, 1H, $J_{3,5}$ 1.7 Hz, $J_{5,6}$ 8.1 Hz, H-5), 3.54 (t, 2H, $J_{1',2'}$ 7.6 Hz, CH_2Br), 3.15 (t, 2H, $J_{1',2'}$7.6 Hz, CH_2Ar), 2.28 (s, 6H, Ac) ppm; ^{13}C-NMR (75.5 MHz, $CDCl_3$) δ 168.2 y 168.1 (2 C=O) 142.0 (C-2), 140.9 (C-1), 137.6 (C-4), 126.7 (C-5), 123.6 y 123.4 (C-3 y C-6), 38.6 (CH_2Ar), 32.0 (CH_2Br), 20.6 (2 COCH_3) ppm; FAB-MS *m/z* 301 ([M+H]$^+$, 45%), 221 ([M−Br]$^+$, 20%; HRFAB-MS *m/z* calcd for $C_{12}H_{14}{}^{79}BrO_4$ ([M+H]$^+$): 301.0075, found 301.0078.

Bis(3,4-diacetoxyphenethyl) diselenide (8)

To a mixture of black selenium (150 mg, 1.901 mmol) in ethanol (11 ml) was added $NaBH_4$ in small portions, under Ar, until the solution color changed to white. The

pH was lowered to 7.0 by adding solid CO_2 in small portions, and then a solution of **7** (520 mg, 1.73 mmol) in THF (11 mL) was added. The mixture was stirred at rt, in the dark under Ar, for 2.5 h. After that, the mixture was concentrated to dryness under vacuum, dissolved in distilled water (75 ml) and extracted with CH_2Cl_2 (75 ml). The aqueous layer was extracted with 2 x 75 mL of CH_2Cl_2. The collected organic fractions were washed with 2 x 75 mL of saturated NaCl, dried over MgSO4 and the solvent was removed under reduced pressure. The residue was purified by column chromatography (EtOAc–hexane 1:20 → EtOAc–hexane 1:3) to give **8** as a yellow solid. Yield 265 mg, 51%; R_F 0.22 (EtOEt–hexane 1:2); mp 98–100 ºC; FT-IR ν_{max} (KBr) 1768, 2914, 2851, 1502, 1427, 1368, 1258, 1192, 1124, 10107, 894, 825, 669 cm^{-1}; ^{1}H NMR (300 MHz, $CDCl_3$) δ 7.11 (d, 2H, $J_{5,6}$ 8.1 Hz, H-5), 7.09 (d, 2H, $J_{2,6}$ 1.6 Hz, H-2), 7.05 (dd, 2H, $J_{5,6}$ 8.1 Hz, $J_{2,6}$ 1.7 Hz, H-6), 3.12 (m, 4H, CH_2Se), 3.03 (m, 4H, CH_2Ar), 2.28 (s, 12H, OAc) ppm; ^{13}C-NMR (75.5 MHz, $CDCl_3$) δ 168.3, 168.2 (2 C=O), 142.0 (C-3), 140.5 (C-4), 139.6 (C-1), 126.6 (C-6), 123.3 (C-2 y C-5), 36.9 (CH_2Ar), 29.9, (CH_2Se), 20.6 (4 CO*C*H_3) ppm; FAB-MS *m/z* 545 ($[M+Na]^+$, 5%); HRFAB-MS *m/z* calcd for $C_{24}H_{26}O_2{}^{80}SeNa$ ($[M+Na]^+$): 545.0688, found: 545.0691.

Bis(3,4-dihydroxyphenethyl) diselenide (**3**)

To a solution of diselenide **8** (150 mg, 0.25 mmol) in CH_2Cl_2–MeOH (7 mL, 1:1) was added a catalytic amount of K_2CO_3, and the mixture was stirred at rt, in the dark under Ar, for 1 h. After that, the pH was lowered with diluted aqueous acetic acid to 6.0, and the mixture was concentrated to dryness at reduced pressure. The residue was purified by column chromatography (CH_2Cl_2→CH_2Cl_2–MeOH 25:1) to give **3** as a yellowish solid. Yield 55 mg, 51 %; R_F 0.39 (CH_2Cl_2–MeOH (10:1); mp 104–108 ºC; FT-IR ν_{max} (KBr) 1281, 3451, 3249, 2918, 1605, 1524, 1439, 1374, 1253, 1179, 1119, 927, 864, 812,

786, 656 cm^{-1}; ^{1}H NMR (300 MHz, CD_3OD) δ 6.68 (d, 2H, $J_{5,6}$ 8.0 Hz, H-6), 6.64 (d, 2H, $J_{3,5}$ 2.0 Hz, H-3), 6.51 (dd, 2H, $J_{3,5}$ 2.0 Hz, $J_{5,6}$ 8.0 Hz, H-5), 3.09 (m, 4H, CH_2Se), 2.87 (m, 4H, CH_2Ar) ppm; ^{13}C-NMR (75.5 MHz, CD_3OD) δ 146.3 (C-2), 144.8 (C-1), 134.0 (C-4), 120.6 (C-5), 111.6 y 111.4 (C-6, C-3), 38.0 (CH_2Ar), 32.3, (CH_2Se) ppm; CI-MS *m/z* 435 ($[M+H]^+$, 2%), 217 ($[M/2]^+$, 30%; HRCI-MS *m/z* calcd for $C_8H_9O_2{}^{80}Se$ ($[M/2]^+$): 216.9776, found: 216.9768.

***N*-(3,4-Dihydroxyphenethyl)-*N'*-phenylselenourea**

To a solution of dopamine hydrochloride (300 mg, 1.58 mmol) and phenyl isoselenocyanate (346 mg, 1.90 mmol, 1.2 equiv) under Ar in MeOH (20 mL) was added Et_3N (220 µL, 1.58 mmol). The mixture was stirred at rt in the dark for 1 h. After that, it was concentrated to dryness and the residue was purified by column chromatography (CH_2Cl_2 → CH_2Cl_2–MeOH 40:1) to give **4** as a yellowish solid. Yield 493 mg, 93%; R_F 0.32 (CH_2Cl_2–MeOH); mp 106-108 ºC; FT-IR ν_{max} (KBr): 1549, 3215, 2362, 1597, 1510, 1447, 1348, 1281, 1187, 1113, 810 cm^{-1}; ^{1}H-NMR (300 MHz, CD_3OD) δ: 7.35 (m, 2H, H-3'y H-5'), 7.23 (m,1H, H-4'), 7.08 (m, 2H, H-2'y H-6'), 6.68 (m, 2H, H-2 y H-5), 6.53 (dd, $J_{2,6}$ 2.0 Hz, $J_{5,6}$ 7.9 Hz, 1H, H-6), 3.81 (t, $J_{CH2,CH2}$ 7.2 Hz, 2H, CH_2NH), 2.78 (t, $J_{CH2,CH2}$ 7.2 Hz, 2H, CH_2Ar) ppm; ^{13}C-NMR (75.5 MHz, CD_3OD) δ: 178.8 (C=Se), 146.4 (C-3), 144.9 (C-4 y C-1'), 131.6 (C-1), 130.8 (C-3' y C-5'), 127.1 (C-4'), 126.1 (C-2' y C-6'), 121.1 (C-6), 117.0 y 116.5 (C-2 y C-5), 49.9 (CH_2NH), 35.2 (CH_2Ar) ppm; FAB-MS *m/z* 337 ($[M+H]^+$, 22%), 359 ($[M+Na]^+$, 25%); HRFAB-MS *m/z* calcd for $C_{15}H_{16}N_2O_2{}^{80}SeNa$ ($[M+Na]^+$): 359.0285, found 359.0275.

***N*-(3,4-Dihydroxyphenethyl)-*N'*-(*p*-tolyl)thiourea** (**5**).

To a solution of dopamine hydrochloride (300 mg, 1.58 mmol) and *p*-tolyl isothiocyanate (283 mg, 1.90 mmol, 1.2 equiv) in MeOH (20 mL) was added Et_3N (220 μL, 1.58 mmol). The mixture was stirred at rt in the dark for 2 h. After that, it was concentrated to dryness and the residue was purified by column chromatography ($CH_2Cl_2 \rightarrow CH_2Cl_2$–MeOH 20:1) to give **5** as a white solid. Yield: 354 mg, 74 %; R_F 0.25 (20:1 CH_2Cl_2–MeOH); mp 151-153 ºC; FT-IR ν_{max} (KBr): 1509, 3351, 3190, 2971, 2922, 2870, 1598, 1542, 1448, 1349, 1276, 1188, 781 cm^{-1}; ^{1}H-NMR (300 MHz, CD_3OD) δ: δ 7.13 (m, 2H, H-3'y H-5'), 6.99 (m, 2H, H-2'y H-6'), 6.67 (d, $J_{5,6}$ 8.0 Hz, 1H, H-5), 6.65 (d, $J_{2,6}$ 2.0 Hz, 1H, H-2), 6.50 (dd, $J_{2,6}$ 2.0 Hz, $J_{5,6}$ 8.0 Hz, 1H, H-6), 3.72 (t, $J_{1',2'}$ 7.0 Hz, 2H, CH_2NH), 2.74 (t, $J_{1',2'}$ 7.0 Hz, 2H, CH_2Ar), 2.31 (s, 3H, CH_3) ppm; ^{13}C-NMR (75.5 MHz, CD_3OD) δ: 181.7 (C=Se), 146.4 (C-3), 144.9 (C-4), 137.3 (C-1'), 136.2 (C-4'), 131.8 (C-1), 131.0 (C-3' y C-5'), 126.0 (C-2' y C-6'), 121.1 (C-6), 116.9 y 116.5 (C-2 y C-5), 47.2 (CH_2NH), 35.2 (CH_2Ar) 21.0 (CH_3) ppm; CI-MS *m/z* 303 ([M+Na]$^+$, 13%); HRCI-MS calcd for $C_{16}H_{19}N_2O_2S$ ([M+H]$^+$): 303.1167, found: 303.1156.

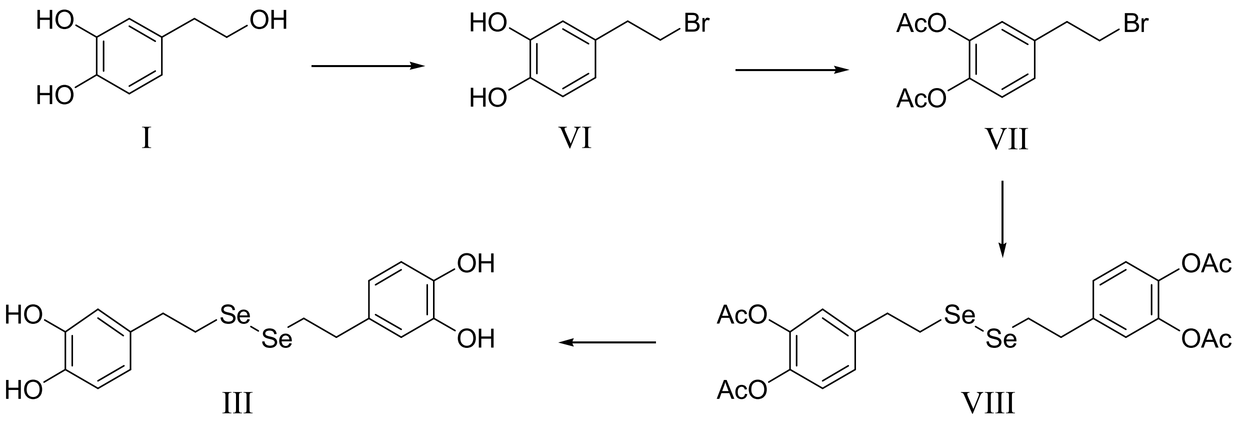

Figure S1: Scheme of synthesis of diselenide of bis-HT.

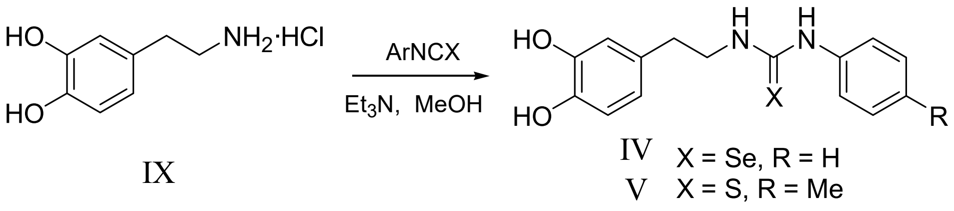

Figure S2: Scheme of synthesis of selenourea and thiourea.
